# Patient and physician related factors of adherence to evidence based guidelines in diabetes mellitus type 2, cardiovascular disease and prevention: a cross sectional study

**DOI:** 10.1186/1471-2296-14-47

**Published:** 2013-04-04

**Authors:** Johanna Fürthauer, Maria Flamm, Andreas Sönnichsen

**Affiliations:** 1 Institute of General Practice and Family Medicine, University of Witten/Herdecke, Alfred-Herrhausen-Str. 50, Witten, 58448, Germany; 2 Institute of General Practice, Family Medicine and Preventive Medicine, Paracelsus Medical University, Strubergasse 21, Salzburg, 5020, Austria; 3 Department of Evidence-based Medicine and Clinical Epidemiology, Danube University, Karl Dorrek-Straße 30, Krems, 3500, Austria

**Keywords:** Non-adherence, Cardiovascular disease, Prevention, Patient adherence, Guideline adherence

## Abstract

**Background:**

Patients do not always receive guideline-adherent therapy, yet little is known about the underlying causes on the patients’ side. We quantified non-guideline-adherent treatment of chronic diseases (diabetes mellitus, hypertension, cardiovascular disease, heart failure, atrial fibrillation) in primary care and analysed the causes from the physician’s as well as the patient’s view.

**Methods:**

With the intention to analyze the frequency and causes of non-guideline-adherent treatment of patients with chronic diseases, we drew a random sample of 124 general practitioners (GP) in Salzburg, Austria, of which 58 (46.8%) participated. In the participating GP surgeries, we consecutively recruited 501 patients with at least one of the target-diseases and checked the guideline conformity of treatment using 9 quality indicators. We then interviewed the patients as well as the general practitioners regarding factors affecting deviation from guideline recommendations.

**Results:**

Of the 501 patients, a total of 1224 quality indicators could be analysed. Non-adherence to guideline recommendations were present in 16.8% (n = 205, 95% CI 14.7 to 18.8%) of all quality indicators. In 61.5% of these cases (n = 126, 95% CI 53.0 to 70.0%) the treatment was wrongly judged as not recommended by the physicians. In 10.2% (n = 21, 95% CI 0 to 23.2%) physicians attributed non-adherence to patient’s non-compliance, and in 10.7% (n = 22, 95% CI 0 to 23.7%) to an adverse drug event, whereas only 5.4% (n = 11, 95% CI 0 to 18.7%) of non-adherence was related to an adverse drug event reported by the patients. Patients were unaware regarding the reason for non-adherent therapy in 64.4% (n = 132, 95% CI 56.2 to 72.6%) of the quality indicators. In 20.0% (n = 41, 95% CI 7.8 to 32.2%) patients regarded a drug as not needed.

**Conclusions:**

Guideline adherence in chronic care was relatively good in our study sample, but still leaving room for improvement. Physicians’ lack of knowledge and patients’ lack of awareness account for about 70% of non-adherence, indicating the necessity to improve physician education, and patient involvement. In about 30% of the quality indicators not fulfilled, non-adherence is due to other reasons like adverse drug events or patients not willing to take a recommended drug.

## Background

To decrease the burden of chronic diseases, treatment guidelines have been developed based on current best evidence from large clinical trials. In Austria, the EbM-Guidelines [1] are most widely used. However, we know from various studies that guidelines are not always applied and followed [2-5]. The barriers to guideline-adherence have been studied quite extensively concerning the physician’s point of view, mainly in qualitative [6-10], but also in quantitative studies. A systematic review of studies addressing physicians’ barriers to guideline adherence, identified physicians’ lack of awareness of a guideline’s existence and lack of familiarity with the guideline as the leading causes of deviation from recommended therapy [11]. Thus ample data exist on the epidemiology of guideline adherence as well as physicians’ barriers to guideline implementation, leading to the assumption that physicians are generally responsible for non-adherence. Based on this assumption, the quality and outcomes framework has been designed in the UK and proven to enhance guideline-adherence substantially [12].

Besides non-adherence due to lack of awareness and lack of familiarity, there may be other reasons for not prescribing a drug which are related to the individual patient, his values, and preferences. Much less is known about patient-related causes of non-adherence to the guidelines. A recent study identified patient ability and patient preferences as potential barriers, based on a survey of GP perceptions [13], but we are not told how patients themselves perceive these barriers, and we could not identify a single study looking at both patient- and physician-related causes of non-adherence at the same time.

We therefore conducted a cross-sectional study in the primary care setting to detect and quantify non-guideline-adherent treatment of chronic diseases, and to quantitatively analyse the causes of non-adherence from the physician’s as well as the patient’s point of view. Since cardiovascular disease represents the major cause of morbidity and death [14], and demographic changes may lead to a further rise in prevalence, we decided to concentrate particularly on these diseases (cardiovascular disease, heart failure, atrial fibrillation) and the most important risk factors (diabetes mellitus type 2 and hypertension).

## Methods

We obtained a complete list of all 200 general practitioners (GPs) under contract with statutory public health insurance in four districts (Salzburg city, Pongau, Tennengau, Flachgau) of the province of Salzburg, Austria. GPs were randomly listed by an electronic randomisation process. Following this order, physicians were asked to participate in the study to obtain a random sample of 58 GPs, corresponding to the study duration of three months (58 consecutive working days from January to April 2011). Each surgery was audited for one day by the principal researcher (JF). On that day, all consecutive patients with at least one of the target diseases or risk factors (arterial hypertension, diabetes mellitus type 2, heart failure, atrial fibrillation, cardiovascular disease) were asked to participate in the study. In Austria, patients can freely choose their GP and usually visit the surgery without arranging appointments. Thus the patient sample represents a random consecutive selection. After signing informed consent, the patients were assessed via a structured case report form (CRF). We collected demographic data, smoking status, patient’s diagnoses regarding chronic diseases, medication and medical data (from the surgery’s patient health record). A detailed description of patient data is presented in Table 1.

**Table 1:** Data collected via case report form (CRF)

**Data collected via case report form (CRF)**		
Demographic data	Age	
	Sex	
	Height	
	Weight	
	Smoking status	
Chronic diseases	Diabetes mellitus type 2	± micro- or macro-vascular complications
	Arterial hypertension	
	Atrial fibrillation	
	Heart failure	(including NYHA-stage)
	Cardiovascular diseases	Myocardial infarction, aortocoronary bypass or stenting, angina pectoris and coronary stenosis* , peripheral arterial occlusive disease, peripheral arterial thromboembolism
	Cerebrovascular diseases	Stroke, transitory ischemic attack, carotid endarterectomy or stenting
Medication	Oral antidiabetics	Metformin
	Antihypertensive drugs	Inhibitors of the renin-angiotensin-aldosterone system (RAAS), diuretics (thiazides, furosemide) calcium channel blockers, beta-blockers
	Platelet aggregation Inhibitors	Acetylsalicylic acid, clopidogrel, prasugrel
	Anticoagulants	Vitamine-K-antagonists
	Lipid lowering therapy	Statins
Medical data (via patient’s health records)	Blood pressure	Singular measurement, multiple self-measurements or 24-h record if available
	HbA_1c_	
	Creatinine level	
	International normalized ratio	In case of oral anticoagulation
	Total serum cholesterol, LDL and HDL levels	

We used 9 quality indicators (QI) based on the EbM-guidelines most commonly applied in Austria [1] to determine guideline-adherence regarding the treatment of the target-diseases mentioned above. The QIs are described in detail in Table 2.

**Table 2:** List of quality indicators (QIs)

**QI**	**Medication**	**Threshold or indication**
**1**	inhibitors of the renin-angiotensin-aldosterone system (RAAS; ACE-I or ARB or Renin-Inhibitors [RI]), calcium channel blockers, β-blockers or thiazides or a combination of these drugs	Arterial hypertension with BP above target (systolic BP >140 mmHg in multiple or 24 h-measurements or >160 mmHg in single measurement)
**2**	Metformin	Diabetes mellitus type 2 (HbA_1c_ >53 mmol/mol (7%))
**3**	β-blocker	Chronic heart failure (any stage)
**4**	RAAS-I	Chronic heart failure (any stage)
**5**	Aldosterone antagonist	Chronic heart failure, NYHA-stage III or IV
**6**	Oral anticoagulation	Atrial fibrillation
**7**	Statin	Any cardiovascular disease (coronary heart disease, cerebrovascular disease, peripheral vascular disease)
**8**	Platelet aggregation inhibitor	Any cardiovascular disease
**9**	β-blocker	History of myocardial infarction

Patients with hypertension were considered to be inadequately treated if they did not reach the target of 140 mmHg in multiple measurements or 24-hour-monitoring, or if a single measurement was above 160 mmHg.

Since we only evaluated cardiovascular diseases as mentioned, it was not possible to evaluate all contraindications for each particular drug. As we collected patients’ creatinine values, it was possible to calculate the GFR and to define a GFR lower than 60 ml/min as an exclusion criterion for metformin therapy. All other more or less weak contraindications, e.g. COPD as a contraindication for β-blockers in the treatment of patients after myocardial infarction, were not specifically obtained and were only recorded as non-specified “contraindications” in our clinical report form (CRF; see Table 3).

**Table 3:** Possible causes of non-adherence to the guidelines evaluated and transferral for analysis

**Patient answer**	**Transferred to**	**Physician answer**	**Transferred to**
I do not know	I do not know	I do not know	Falsely not indicated GP
I do not need it	I do not need it	Not indicated	
I do not want it	I do not want it	Noncompliance	Noncompliance
		Avoidance of polypharmacy	Avoidance of polypharmacy
		Specialist did not prescribe it	Falsely not indicated s/h*
I do not take it any more	Adverse drug event	Contraindication	Contraindication
	Falsely discontinued^†^	Patient does not take it anymore	Adverse drug event
			Falsely discontinued^†^
	*(or one of the above)*		*(or one of the above)*
Other	*Allocation to one of the above*	Other	*Allocation to one of the above*

* Falsely not indicated specialist / hospital (when a certain therapy was not initiated or recommended by specialists or physicians in the hospital, and the GP adhered to this recommendation even though in fact there was an indication for the drug).

^†^ Defined as a stop of treatment triggered by the physician despite guidelines recommending a continuation of the therapy (e.g. discontinuation of statin therapy after reaching target values for LDL-cholesterol which then rose above target again).

We then interviewed the patient and the physician independently about underlying reasons for non-adherent treatment, using a structured CRF. The reasons given by patients and physicians are presented in Table 3. The category “other” was offered but it was possible to transfer each answer to one of the other offered causes. All data were pseudonymised and recorded in pre-specified case report forms and then transferred to IBM® SPSS® Statistics19.0 for further analysis.

The study was carried out in compliance with the Declaration of Helsinki and with Austrian data protection legislation. Ethics approval has been obtained from the ethics committee of the federal state of Salzburg, Austria.

## Results

We had to randomly select 124 of the 200 GPs eligible to obtain a sample of 58 GPs willing to participate (response rate 46.8%). In these 58 GP surgeries 526 consecutive patients were invited to participate in the study. 501 of these patients (95.3%) gave informed consent and were finally included in the analyses, corresponding to a mean of 8.6 ± 5.3 (SD) patients per surgery. These patients had a total number of 922 target diseases with a total number of 1224 QIs analysed (three QIs for heart failure, three QIs for cardiovascular disease, and one QI for each of the other diseases). Descriptive data of all patients are depicted in Table 4.

**Table 4:** Descriptive data of all patients (mean ± standard deviation)

**Descriptive data of all patients**		
Male total n	251	(50.1%)
Male smokers (%)	15.1	
Female total n	250	(49.9%)
Female smokers (%)	9.6	
Age (years ± SD)	69.5	± 10.9
BMI (kg/m^2^ ± SD)	28.1	± 4.8

**Figure 1:**
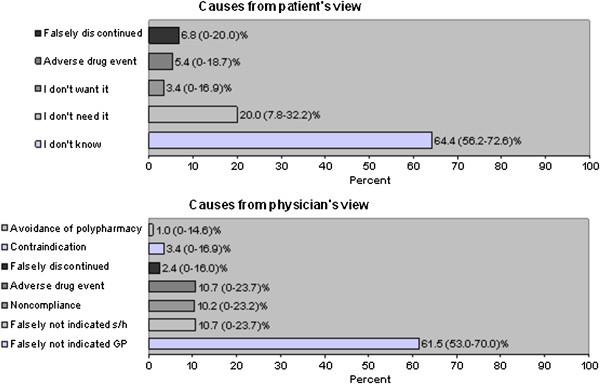
**Causes of deviations from the guidelines.** Percentages are percent of all Quality Indicators analyzed. Patient’s view: “Falsely discontinued” is defined as a stop of treatment triggered by the physician despite guidelines recommending a continuation of the therapy (e.g. discontinuation of statin therapy after reaching target values for LDL-cholesterol). Physician’s view: “Falsely not indicated general practitioner (GP)” signifies that the GP never started therapy due to e.g. a knowledge gap and the drug in fact was indicated. “Falsely not indicated specialist / hospital (s/h)” signifies that a certain therapy was not initiated or recommended by specialists or physicians in the hospital and the GP adhered to this recommendation when in fact there was an indication for the drug.

The prevalence of the targeted chronic diseases within our cohort and the percentage of patients not treated according to the guidelines using the QIs defined above are shown in Table 5. In 185 (36.9%) of all patients, at least one QI was not fulfilled. Overall, guideline adherence was not given in 16.8% (95% CI 14.7 to 18.8%) of all QIs. The distribution of possible causes of non-adherent therapy is shown in Figure 1.

**Table 5:** Diagnoses with numbers of QIs and QIs revealing non-adherence to the guidelines

**Diagnosis**	**n of cases with diagnosis (number of QIs)**	**n of QIs revealing non-adherent treatment**	**% QIs revealing non-adherent treatment (95% CI)**	
**Arterial hypertension**	424 (424)	69	16.3	(12.8-19.9)
**Diabetes mellitus type 2**	174 (174)	8	4.6	(1.5-7.7)
**Heart failure**	41 (89)	27	30.3	(20.8-39.9)
β-Blocker	41 (41)	15	36.6	(21.8-51.3)
RAAS-I	41 (41)	8	19.5	(7.4-31.6)
Aldo-A in NYHA III/IV	7 (7)	4	57.1	(20.5-93.8)
**Atrial fibrillation**	106 (106)	15	14.2	(7.5-20.8)
**Cardiovascular disease**	177 (431)	86	20.0	(16.2-23.7)
Statin	177 (177)	53	29.9	(23.2-36.7)
Platelet aggregation inhibitors	177 (177)	12	6.8	(3.1-10.5)
β-blocker after myocardial infarction	77 (77)	21	27.3	(17.3-37.2)
Total*	922 (1224)	205	16.8	(14.7-18.8)

* The number of diagnoses exceeds the number of patients as many subjects had more than one of the target diagnoses.

We performed a detailed analysis of underlying causes for non-guideline-adherent treatment referring to each target diagnosis and QI. The results of this analysis are presented in Table 6.

**Table 6:** Causes of deviation from guidelines for every drug and diagnosis in %

**Causes of deviation from guidelines from patient’s view**									
**n**	**69**	**8**	**15**	**8**	**4**	**15**	**53**	**12**	**21**
**Reason (in %)**	**Anti-HT**	**Metformin**	**HF: βB**	**HF: RAAS-I**	**HF: AldoA**	**AF: OAK**	**CVD: statin**	**CVD: antipltlt**	**MCI: βB**
I don’t know	91.3	50.0	60.0	62.5	75.0	66.7	30.2	50.0	76.2
I don’t need it	2.9	25.0	26.7	37.5	25.0		45.3	25.0	9.5
I don’t want it		12.5				13.3	7.5		
Adverse drug event	4.3		13.3			6.7	7.5		4.8
Falsely discontinued	1.4	12.5				13.3	9.4	25.0	9.5
**Causes of deviation from guidelines from physician’s view**									
**n**	**69**	**8**	**15**	**8**	**4**	**15**	**53**	**12**	**21**
**Reason (in %)**	**Anti-HT**	**Metformin**	**HF: βB**	**HF: RAAS-I**	**HF: AldoA**	**AF: OAK**	**CVD: statin**	**CVD: PAI**	**MCI: βB**
Falsely not indicated GP	78.3	37.5	80.0	62.5	50.0	33.3	56.6	33.3	52.4
Falsely not indicated s/h	8.7	12.5	6.7	25.0	25.0	6.7	7.5	8.3	23.8
Noncompliance	2.9	25.0				20.0	17.0	25.0	9.5
Adverse drug event	8.7	25.0	6.7	12.5	25.0	6.7	11.3	25.0	4.8
Falsely discontinued	1.4		6.7			6.7	3.8		
Contraindication						26.7		8.3	9.5
Avoidance of polypharmacy							3.8		

Summation does not add up to 100% due to rounding error.

Anti-HT: antihypertensive medication (RAAS-inhibitor or thiazide or calcium channel blocker or β-blocker).

Metformin: metformin in diabetes mellitus type 2 patients.

HF = heart failure.

βB: beta adrenoceptor blocker.

RAAS-I: inhibitor of the renin-angiotensin-aldosterone system.

AldoA: aldosterone antagonist.

AF = atrial fibrillation.

OAK: oral anticoagulation.

CVD = cardiovascular disease.

PAI: platelet aggregation inhibitor (acetylsalicylic acid or clopidogrel/prasugrel).

MCI = myocardial infarction.

Arterial hypertension was present in 424 patients (84.6%). 76 patients (17.9%) had blood pressure measurements above target (QI 1). A combination of up to four antihypertensive drugs is recommended to reach optimal blood pressure levels. Adequate combination therapy of four antihypertensive drugs was prescribed in only 7 of the patients with blood pressure above target (9.2%). The causes of not fulfilling QI 1 in the remaining 69 patients (90.8%) are listed in Table 6. Nineteen (27.5% of all) of them were treated with a three-drug combination.

The study cohort included 174 patients with diabetes mellitus type 2, of whom 64 (36.8% of all) did not receive metformin (QI 2). Thirty-two had a contraindication for metformin (glomerular filtration rate [GFR] </=60 ml/min, using the Cockroft-Gault-formula). We could not obtain a creatinine value for two of the patients. Nineteen of the remaining 30 patients had an HbA_1c_ value of less than 53 mmol/mol (7%), and for three patients, we could not obtain an HbA_1c_ value. All in all, QI 2 was not fulfilled in only eight (4.6%) of all patients with diabetes mellitus type 2. The causes of deviation from the guideline are listed in Table 6.

We found 41 patients with heart failure in our cohort, 15 (36.6% of all) did not receive any β-blocker (QI 3) and 8 (19.5% of all) were not treated with an RAAS-inhibitor (QI 4), as would have been recommended. Seven (17.1% of all) patients had a NYHA stage of III or more. The NYHA stage was not definable in 3 patients, so QI 5 was not fulfilled only in 4 patients (9.8% of all). The causes for guideline deviation are listed in Table 6.

Atrial fibrillation was present in 106 patients, of whom 15 (14.0% of all) were not treated with oral anticoagulants as defined in QI 6. The reasons for non-adherence are presented in Table 6.

The diagnosis of any cardiovascular disease was present in 177 patients. Fifty-three (29.9%) patients were not prescribed any statin (QI 7). Two of them were treated with a fibrate only, and one with ezetimib only. The reasons for not receiving a statin or any lipid-lowering treatment as defined in QI 7 are listed in Table 6.

Furthermore, 53 (29.9%) of the patients with cardiovascular disease did not take platelet aggregation inhibitors (acetylsalicylic acid or clopidogrel or prasugrel) in accordance with QI 8. As a meta-analysis of randomised trials showed that the combination of platelet aggregation inhibitors and oral anticoagulants in patients with atrial fibrillation does not have any additive benefit but carries a higher risk for bleeding [15], we considered oral anticoagulants an acceptable reason to discontinue antiplatelet-therapy. This was the case in 41 patients, so only 12 (6.8%) were not treated as recommended by the guidelines (QI 8). The causes of non-adherence to the guidelines are listed in Table 6.

A history of myocardial infarction was present in 77 of the 177 patients with cardiovascular disease, and 21 (27.3% of all patients with myocardial infarction) of them were not on β-blocking treatment as defined in QI 9. The causes for non-adherence are listed in Table 6.

## Discussion

We found obvious deficits in care regarding guideline adherent drug therapy for hypertension, diabetes mellitus type two, heart failure, atrial fibrillation and secondary prevention in cardiovascular diseases. About a sixth of all quality indicators in our study were not fulfilled according to current guideline recommendations. In more than half of these quality indicators the patients did not know why they were not prescribed a particular drug, thus making us look at the physician as the one responsible for non-adherence. The most frequent reason for physicians to deviate from guideline recommendations was that they falsely assumed that a certain prescription was not indicated or necessary.

There are several possible explanations for the fact that the treatment of patients is not always consistent with evidence based recommendations. According to our study, the most important cause appears to be the physician not providing a particular treatment. This may be due to physicians’ lack of awareness regarding the existence of a guideline, or lack of familiarity with a guideline, as has been shown by Cabana et al. [11]. However, non-adherence may also be caused by a deliberate decision to counteract the guideline with which the physician may not agree, in general or for a particular patient.

Even though the GP appears to be the main cause of non-adherence to the guidelines, our study clearly shows that other reasons are involved in at least one third of all quality indicators.

Of the non-GP-related causes, adverse drug events and non-compliance appear to be the most important. In chronic care, GPs are confronted with the problem that they have to keep the patient compliant over a long period of time, and that any drug treatment has to match up to other health goals and is influenced by psychosocial problems [16]. Chapman and co-authors found a sharp decline in drug-adherence to lipid and blood pressure lowering drugs to only 36% within one year, with the greatest drop occurring in the first three months [17]. The factors determining compliance are manifold: Health education appears to play a crucial role, but other patient specific factors like race, ethnicity, or education are also important [18]. Sometimes patients do not seem to be aware of their illness, or they accept their chronic disease symptoms as normal, e.g. as a result of ageing. Thus, more than half of the patients with heart failure reported their health to be good even though nearly half of them could not walk a quarter of a mile [5]. Another problem may be that many drug effects in cardiovascular prevention are hardly noticeable to the patient so that the importance of the medication remains unrecognized. Patients’ fears of adverse drug reactions certainly also play a role in non-adherence to recommended treatments. Moreover, insufficient communication including incomplete patient’s history taking, conflicting information, neglected disagreements, or a disturbed relationship between the patient and the physician may cause non-adherence or non-compliance [19].

The fact that about two thirds of the patients did not know why they do not receive a recommended drug points out a significant information deficit. While we would not expect all patients to wish to be informed about treatment options, there appears to exist sufficient evidence that most patients would prefer to be involved in evidence based treatment decisions [20]. From our study we cannot distinguish whether the information deficit is due to a lack of communication between physician and patient, or to the patient not wanting to be involved. Looking at one of the leading models for shared decision making it takes both the physician’s willingness to share information as well as the patient’s desire to be informed [21], and we conclude from our study that it seems unlikely that about two thirds of the patients do not want to know about guideline-adherent, evidence-based treatment choices.

About 20% of the patients in our study stated that they do not need a drug they should in fact receive. This might reflect certain knowledge deficits regarding present diseases or risk factors.

Although our study reveals important insights regarding the causes of non-adherence to guideline recommendations, some limitations have to be considered. A major weakness of our study is that the sample size is fairly small. This especially limits the explanatory power of the detailed analysis of single diseases, and even the power of the combined data presented in Figure 1 cannot be considered sufficient due to large confidence intervals reaching zero on the left side.

Even though we chose a random sample of physicians, our study may be biased by a response rate of only 50% on the physician level. On the other hand, this response rate is quite usual in studies involving primary care physicians. The systematic review of Cabana et al. reports a median response rate of 54.5% for studies investigating physicians’ lack of awareness as one of the barriers to guideline adherence [11].

It may be assumed that rather motivated physicians who already provide higher quality service to their patients are more likely to participate in a study of quality analysis. But as our main goal in this study was not to quantitatively analyse non-adherence, but rather differentiate the possible reasons for non-adherence, we believe our data to be quite representative.

We visited each general practitioner only one day and included only patients that consulted their doctor at that time. This may bias our results as well, as one may assume that less compliant patients tend to visit their physician only at rare intervals. We tried to overcome this problem by also including patients coming to the surgery only to pick up a prescription.

We used a narrow set of only process quality indicators to judge guideline adherence in drug therapy. This may also be considered a weakness of our study. In diabetes mellitus type 2, for instance, it has been demonstrated that there is insufficient evidence for many widely used quality indicators regarding their predictive power for clinically relevant outcomes [22]. This appears to be a general problem of using quality indicators, and is not specific to our study.

Last not least our structured interview technique could only obtain categorical data on possible reasons for non-adherence to evidence based guidelines. An in-depth qualitative analysis of individual patients’ reasons for not taking a drug or physicians’ reasons for not prescribing it would be highly desirable and warrants further research.

The strengths of our study are that we examined a representative sample of consecutive patients from both rural and urban areas, and we included all major diseases affecting the cardiovascular system. So far very little is known about the causes of non-adherence to guideline recommended therapy on the patient’s side, and thus this study provides data inspiring further research to improve guideline-adherence in chronic care.

## Conclusion

Overall, about 15% of all QI-cases examined in this study were not treated according to the guidelines, and in 72% of these a physician, either GP or specialist, judged a drug falsely as not guideline recommended. In about the same proportion of cases, the patients stated that they did not know why they did not receive a drug recommended by the guidelines, indicating information deficits on the patient’s side. Lack of awareness, lack of knowledge, overlooking values above target, or lack of communication, are possible underlying causes. On the other hand, about 30% of the QIs not fulfilled are due to various other reasons.

Our study points out that the improvement of guideline adherent care is far more than getting physicians to follow the guidelines. Patient information, patient involvement and the physician’s willingness to inform and involve the patient are just as important. Finally, our study makes clear that 100% guideline-adherent care cannot be achieved due to e.g. adverse drug reactions and contraindications. Also, the deliberate decision of the patient not to take a particular drug must be respected.

## Abbreviations

BP: Blood pressure; ACE-I: Angiotensin-converting enzyme inhibitor; ARB: Angiotensin receptor blocker; GP: General practitioner; CRF: Case report form; QI: Quality indicator; RAAS: Renin-angiotensin-aldosterone system; RAAS-I: Inhibitor of the renin-angiotensin-aldosterone system; RI: Renin inhibitor

## Competing interests

The authors declare that they have no competing interests, that there are neither support from any organisation for the submitted work, nor financial relationships with any organisations that might have an interest in the submitted work, nor other relationships or activities that could appear to have influenced the submitted work.

## Authors’ contributions

JF was the main researcher who collected the data, did the analysis of the data, and did the main research and writing. MF contributed to formatting, research and writing. AS designed the study and the CRF together with JF and participated in the writing process. All authors read and approved the final manuscript.

## Pre-publication history

The pre-publication history for this paper can be accessed here:

http://www.biomedcentral.com/1471-2296/14/47/prepub
